# The Involvement of Sortilin/NTSR3 in Depression as the Progenitor of Spadin and Its Role in the Membrane Expression of TREK-1

**DOI:** 10.3389/fphar.2018.01541

**Published:** 2019-01-08

**Authors:** Jean Mazella, Marc Borsotto, Catherine Heurteaux

**Affiliations:** CNRS, UMR 7275, Institut de Pharmacologie Moléculaire et Cellulaire, Université Côte d’Azur, Valbonne, France

**Keywords:** sortilin, TREK-1 channel, depression, protein complex, spadin

## Abstract

The molecular identification of sortilin, also called neurotensin receptor-3, from three different biochemical approaches already predicted the involvement of the protein in numerous biological and cellular functions. The first important observation was that sortilin is synthesized as a precursor that is converted to a mature protein after cleavage by the protein convertase furin in late Golgi compartments. This maturation leads to the formation of a 44 amino acid peptide, the propeptide (PE). The release of this peptide when matured sortilin reached the plasma membrane remained to be demonstrated. Sortilin has been also shown to be shedded by matrix metalloproteases releasing a large extracellular fragment identified as soluble sortilin. Therefore, sortilin has been shown to interact with several proteins and receptors confirming its role in the sorting of cellular components to the plasma membrane and/or to the lysosomal pathway. Interestingly, sortilin physically interacts with the two pore domain potassium channel TREK-1 and the PE as well as its synthetic analog spadin is able to block the activation of TREK-1 highlighting their role in the depression pathology. The present review describes the advance of research that led to these results and how both the soluble form of sortilin and the sortilin-derived PE have been detected in human serum and whose levels are affected in patients with major depressive disorder (MDD). The use of spadin as an antidepressant and the further role of soluble sortilin and of sortilin-derived PE as potential biomarkers during depression statement and/or remission of the pathology are considered and discussed in this review.

## Introduction

From the family of Vps10p-domain receptors including Sortilin, SorLa and SorCS1-3 (for review see Hermey, 2009; Glerup et al., 2014), sortilin is certainly the type I transmembrane receptor protein bearing the most wide range of cellular, membrane and extracellular functions. Sortilin is expressed in neuronal and non-neuronal cells from various important brain areas (Petersen et al., 1997; Sarret et al., 2003). It is also present in non-neuronal cells from numerous peripheral organs including liver, pancreas, heart, lung, skeletic muscle, adipose tissue, and immune system (Petersen et al., 1997; Wilson et al., 2014). The developmental expression of sortilin was also analyzed in mice (Hermans-Borgmeyer et al., 1999). As soon as the discovery of sortilin and its molecular identification from three different biochemical approaches like receptor associated protein (RAP) and neurotensin (NT) affinity chromatography (Petersen et al., 1997; Mazella et al., 1998), and immunoisolation from the glucose transporter Glut4-containing vesicles (Lin et al., 1997; Morris et al., 1998), the involvement of sortilin in several important biological functions has been predicted (Mazella, 2001).

The properties of sortilin, also called NT receptor-3, appear complex. It was necessary to co-transfect the protein convertase furin with sortilin to obtain a receptor with a high affinity for NT (Mazella et al., 1998). The maturation of sortilin by furin that leads to the release of a 44 amino acid propeptide (PE) and a functional receptor was confirmed by a nice work using mutagenesis approach (Munck Petersen et al., 1999). It is crucial to note that PE is also able to bind sortilin with a high affinity. The multifunctional role of sortilin has been successively demonstrated by a series of studies identifying, in addition to NT (Mazella et al., 1998) several other ligands for this receptor including the Lipoprotein Lipase (Nielsen et al., 1999), the pro-form of the Nerve Growth Factor, pro-NGF (Nykjaer et al., 2004) and of the Brain Derived Neurotrophic Factor, pro-BDNF (Teng et al., 2005). Surprisingly and interestingly, all these ligands bind to sortilin within the same binding site formed by a tunnel of a ten-bladed beta propeller domain (Quistgaard et al., 2009). Interestingly, two nice works demonstrated that low-pH recovered during endocytosis of sortilin with its ligands induces conformational change and dimerization of the protein that triggers ligand release by collapsis of the binding domain (Januliene et al., 2017; Leloup et al., 2017). However, binding competition of these ligands to sortilin remains pharmacologically difficult to understand since their active sequences are totally different (i.e., the hexapeptide C-terminal part of NT and the pro-domain of NGF).

## Sortilin and its Preferred Partner Trek-1 in Depression

From the multiple functions linked to multiple cellular protein partners (intracellular or membrane bound) of sortilin, we will only discuss in this chapter on receptor or channel proteins that are associated with sortilin within the cell surface and will focus on its crucial role in the sorting of the 2 pore potassium channel TREK-1 (for Twik Related Potassium Channel 1).

The first demonstration of a role of co-receptor for sortilin was shown in the human adenocarcinoma cell line HT29 where NT receptor-1 (NTSR1) and sortilin form a membrane protein complex able to internalize together and modulate the cellular NT signaling (Martin et al., 2002). In agreement with its ability to bind pro-NGF and pro-BDNF, it has been demonstrated that sortilin interacts with their respective receptors p75NTR (Nykjaer et al., 2004; Teng et al., 2005) and Tyrosine protein kinase receptor B (TrkB) (Fauchais et al., 2008; Akil et al., 2011). The interaction of sortilin with p75NTR generally leads to neuronal cell death through the pro-domain of neurotrophins (Nykjaer et al., 2004; Teng et al., 2005). By contrast, in peripheral cells or organs, the interaction of sortilin with p75NTR leads to renal cancer cell survival (De la Cruz-Morcillo et al., 2016) as well as B cell survival (Fauchais et al., 2008) whereas the complex between sortilin and TrkB is involved in colorectal cancer cell survival (Akil et al., 2011). In pancreatic beta cells, the association of sortilin with the NT receptor-2 (NTSR2) appears crucial to trigger the anti-apoptotic NT action (Beraud-Dufour et al., 2009).

With the aim to try to understand why both mice in which TREK-1 (*kcnk2*) or sortilin (*sort1*) gene was deleted, presented a similar phenotype of resistance to depressive-like behavior during resignation tests [Forced swimming test (FST) and tail suspension test (TST)], the putative physical interaction between the two proteins was investigated. TREK-1 belongs to the family of two pore-domain potassium channels (K2P) and plays, under the formation of dimers, a crucial role in neuroprotection, pain, anesthesia and depression (Honore, 2007; Borsotto et al., 2015). The activity of TREK-1 channels is regulated by numerous factors like temperature, membrane stretch, pH and by polyunsaturated fatty acids and volatile anesthetics (Vivier et al., 2016). In the field of mood disorders, the deletion of the TREK-1 gene (**kcnk2*^-/-^)* in mice leads to animals that have an antidepressant phenotype (Heurteaux et al., 2006). Therefore, TREK-1 became an interesting target for the research of molecules capable to block its activity. As regards sortilin, preliminary behavioral experiments performed in mice in which the gene coding for sortilin has been inactivated (*Sort1^-/-^)* indicated a trend to be less resigned than wild-type mice in two tests commonly used to characterize antidepressant molecules (FST and TST). The resulting hypothesis from these observations was that the presence of sortilin could be crucial for the correct sorting of the TREK-1 channel. In the absence of sortilin, TREK-1 is not correctly addressed to the plasma membrane leading in FST and TST to similar phenotypes as observed with KO mice.

**FIGURE 1:**
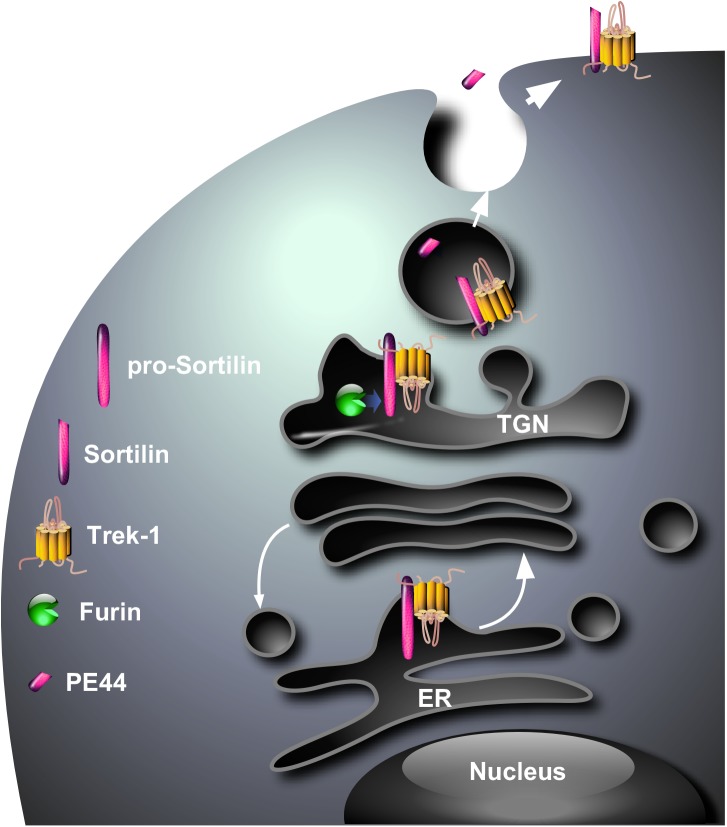
Hypothetical model for the cellular sorting of the complex sortilin/TREK-1. The complex that is probably formed in endoplasmic reticulum (ER) translocates to the *Trans*-Golgi Network where the PE is cleaved by the protein convertase furin. The complex sortilin/TREK-1 can be addressed *via* constitutive secretory vesicles from the TGN to the plasma membrane where the PE (PE44) is released in the circulation.

Since one of the major functions of sortilin is to address numerous proteins from intracellular compartments to the plasma membrane or lysosomes (Lefrancois et al., 2003; Kandror and Pilch, 2011), the question was “Are sortilin and TREK-1 physically associated ?” and, if yes “ Is sortilin involved in the sorting of TREK-1 ?” In this way, we have demonstrated using cross immuno-precipitation that a protein complex existed between sortilin and TREK-1, both endogenously co-localized in same neurons (Mazella et al., 2010). Moreover, the expression of the TREK-1 channel at the plasma membrane of COS-7 cells is strongly increased when the channel is co-expressed with sortilin (Mazella et al., 2010). The biochemical determination of the importance of the sortilin/TREK-1 complex in the depressive-like behavior has been definitively confirmed by the demonstration that the brain of *Sort1*^-/-^ mice presents an altered TREK-1 function, essentially due to a lower expression of the channel at the neuronal plasma membrane (Moreno et al., 2018). The resulting consequence of TREK-1 dysfunction is an increased activity of dorsal raphe nucleus neurons and a decreased depressive-like behavior of *Sort1*^-/-^ mice (Moreno et al., 2018) very similar to that observed in *Kcnk2*^-/-^ mice (Heurteaux et al., 2006). These observations strongly suggest that sortilin is necessary for the correct sorting of TREK-1 to the cell surface (Figure 1). The remaining question is “Does the sortilin/TREK-1 complex exists only intracellularly or only at the plasma membrane or both?” Although not directly demonstrated, the complex is likely formed in the endoplasmic reticulum, maintained at the level of the *Trans-*Golgi Network in which pro-sortilin is matured by furin (Figure 1) and the complex TREK-1/sortilin in vesicles merges with the plasma membrane with the release of PE.

## The Discovery of Spadin as a Potent Antidepressant in Mice

The first role of PE was observed on the neurotensinergic system with the demonstration that the peptide antagonized the human microglial migration induced by NT (Martin et al., 2003) as well as the NT-activated expression and release of cytokines/chemokines from a murine microglial cell line (Dicou et al., 2004). Both human and murine microglial cells do not express NTSR1 and NTSR2, but only the NTSR3/sortilin on which PE displays an antagonistic role on NT-induced microglia migration and cytokine release. These results suggest that PE could act as an anti-inflammatory regulator in the brain.

From the fact that NT and PE are able to bind matured sortilin, the existence of the complex between sortilin and TREK-1 prompted us to test both peptides on the channel activity. On COS-7 cells expressing TREK-1, when the channel was activated by arachidonic acid (AA), PE efficiently decreased by 70–80% the AA-induced channel activation. NT was without effect (Mazella et al., 2010). Consequently, the inhibition of TREK-1 by PE and its shorter designed analog spadin induce a potent antidepressant effect in numerous depressive-like behavior tests These results confirmed that targeting TREK-1 channels by a peptide designed from the endogenous PE could represent a new concept in the antidepressant drug design. These observations were reinforced by the rapid onset of action of spadin on neurogenesis (4 days for spadin *vs* 21 days for fluoxetine) (Mazella et al., 2010) and by the efficient spadin-mediated activation of synaptogenesis and spinogenesis (Devader et al., 2015). Moreover, the absence of TREK-1-related side effects of spadin on cardiac function, pain, and glucose homeostasis reinforced the possibility to develop spadin or spadin analogs up to clinical trials (Moha Ou Maati et al., 2012). Accordingly, with the goal to obtain a molecule with a better affinity for TREK-1, structure-function relationships studies allowed to define a shorter active peptide from PE, the sequence 22–28, called mini-spadin, and its more stable modified analogs G/AVSWGLR and Biotin-AVSWGLR (Djillani et al., 2017). These peptides display an affinity for TREK-1 40–350 times better than spadin itself and keep efficient antidepressant activities as recently reviewed (Djillani et al., 2018).

## The Sortilin-Derived Peptides as Biomarkers of Depression State and Remission

To the probability of PE and its derivatives to be released in the circulation, the release of a soluble form of sortilin (s-sortilin) by matrix metalloproteases-induced shedding from the plasma membrane (Navarro et al., 2002) represented a new fragment of sortilin that can be found in the circulation. Indeed, the shedding of sortilin is essentially mediated by the metalloprotease ADAM10 (for a desintegrin and metalloprotease 10) (Navarro et al., 2002; Evans et al., 2011) and to a lesser extend by ADAM17 (also called TACE for tumor necrosis factor alpha-converting enzyme) (Hermey et al., 2006). These characteristics prompted researchers to develop dosing techniques to measure seric or plasmatic contents of sortilin fragments (i.e., PE or s-sortilin) in healthy controls and in patients suffering from major depressive disorder (MDD).

The first observation was made by measuring the serum levels of s-sortilin that are increased in association with depression and correlated with the serum levels of BDNF and VEGF (Buttenschon et al., 2015). However, although the interesting result obtained above, investigation of serum content of s-sortilin in response to antidepressant treatment indicated that no significant change was observed (Buttenschon et al., 2017; Table 1). The authors concluded that this analysis does not point toward sortilin as a state-dependent biomarker.

**Table 1:** Seric concentration of s-sortilin and sortilin-derived peptide in healthy controls, MDD before and after AD treatment or ECT.

Seric [peptide]	Healthy controls	MDD/controls	12 week AD treatment	AD treatment-resistant	AD treatment-resistant+ECT
S-sortilin^∗^	Basal level (*n* = 216)	Increased (*n* = 152)	Remains increased (*n* = 56)	n.d.	n.d.
Sortilin-derived peptide PE^∗∗^	Basal level (*n* = 49)	Decreased (*n* = 37)	Restores basal level (*n* = 37)	Basal level (*n* = 49)	Increased (R) (*n* = 35)

*^∗^From Buttenschon et al. (2015, 2017). ^∗∗^From Devader et al. (2017) and Roulot et al. (2018). MDD, major depressive disorder; AD, antidepressant; R, responders; ECT, electroconvulsive therapy; n.d., not determined.*

Recently, serum sortilin-derived PE was shown to be decreased in MDD patients and interestingly, pharmacological antidepressant treatment restore normal serum PE level in treatment-responder patients (Devader et al., 2017; Table 1). In addition, treatment-resistant depressed patients who present no change in the amount of sortilin-derived PE and who are further treated by electroconvulsive therapy show a strong increase of sortilin-derived PE 1 month after the therapy (Roulot et al., 2018; Table 1). Here again, only patients who respond to electroconvulsive therapy present a significant increase in PE levels. Altogether, these results indicate that sortilin-derived PE could serve as a marker of the depression state and also as an indicator of the remission of the pathology. Measurement of serum sortilin-derived PE levels in addition to other already known endogenous seric markers for depressive state like BDNF (Bocchio-Chiavetto et al., 2006; Hu et al., 2010; Molendijk et al., 2014) and VEGF (Minelli et al., 2011; Stelzhammer et al., 2013) could assist psychiatrics in the diagnosis of antidepressant response efficacy and therapy success.

Both s-sortilin and sortilin-derived PE serum levels are modified in MDD patients. But the amount of s-sortilin is increased whereas the level of sortilin-derived PE is decreased in patients, although both PE and s-sortilin come from the same protein pro-sortilin. This discrepancy is likely due to the fact that the PE formation is performed intracellularly by the intervention of the protein convertase furin in the *Trans-*Golgi Network (Munck Petersen et al., 1999), whereas the release of s-sortilin by shedding is processed by the protein kinase C-dependent activation of the matrix metalloproteases (Navarro et al., 2002). These two systems are likely differently regulated during the multiple peripheral and central dysregulations observed in MDD patients.

## Conclusion and Perspectives

The important information to retain from these works is that all the situations that decrease TREK-1 function, as observed in *kcnk2*^-^*^/^*^-^ and in *Sort1*^-^*^/^*^-^ mice or after inhibition by spadin and its analogs, lead to a decrease of depressive-like behavior. The different mechanisms involved in this common phenotype from different experimental approaches remain to be deepened, although partly documented.

The further use of spadin and/or spadin analogs in clinical trials is currently in progress and could lead to the development of a treatment for MDD patients, at least for treatment-resistant depressed patients. Moreover, the determination of serum levels of sortilin-derived peptide and of the soluble form of sortilin could not only increase the efficiency of the quality of diagnosis for psychiatrists and also could serve to confirm the remission of the pathology.

Although most of the research works with spadin have been performed in our laboratory, the « spadin concept » targeting TREK-1 has been validated by several other international laboratories in the field of depression, anxiety and stress (Bista et al., 2012; Zhang et al., 2015; Lengyel et al., 2016; Vallee et al., 2016; Qi et al., 2018).

## Author Contributions

All authors listed have made a substantial, direct and intellectual contribution to the work, and approved it for publication.

## Conflict of Interest Statement

The authors declare that the research was conducted in the absence of any commercial or financial relationships that could be construed as a potential conflict of interest.
